# Subtelomeric elements provide stability to short telomeres in telomerase-negative cells of the budding yeast *Naumovozyma castellii*

**DOI:** 10.1007/s00294-025-01325-w

**Published:** 2025-09-03

**Authors:** Rishi K. Jaiswal, Teresa Garibo Domingo, Héloïse Grunchec, Komudi Singh, Mehdi Pirooznia, Eran Elhaik, Marita Cohn

**Affiliations:** 1 Department of Biology, Lund University, Sölvegatan 35, SE-223 62 Lund, Sweden; 2 Bioinformatics and Computational Core Facility, NIH, Bethesda, MD USA

**Keywords:** Telomere, Genome integrity, Alternative lengthening of telomeres (ALT), Telomerase-independent telomere maintenance, Subtelomeres, Budding yeast* Naumovozyma castellii*

## Abstract

Telomerase plays an important role in sustaining eukaryotic linear chromosomes, as elongation of telomeres is needed to counterbalance the shortening occurring in each replication round. Nevertheless, in telomerase-deficient cells, Alternative Lengthening of Telomeres (ALT) pathways can maintain telomeres by employing recombination-based mechanisms. In the budding yeast *Naumovozyma castellii*, effective activation of the ALT pathway leads to bypass of senescence and supports long-term growth. We found that telomere structures in *N. castellii* ALT cells are stably maintained at a shortened uniform length over extensive numbers of generations. This is correlated to the spreading of a subtelomeric sequence, TelKO element, to all telomeres. Genome sequencing of the wild-type strain revealed variants of the TelKO element, differing in their lengths, and separate ALT strains are maintained by spreading of distinct TelKO element variants. Although short uniform telomere structures are predominant, sporadic telomere lengthening events occur by addition of long repeated arrays of TelKO elements. The telomere-binding protein Rap1 can bind to TelKO sequences in vitro, indicating a functional role of TelKO elements in providing stability to shortened ALT telomeres. Our results suggest that stable maintenance and telomere functionality may be achieved by incorporating the distal subtelomeric TelKO sequences into the telomeric chromatin cap.

**Supplementary Information:**

The online version contains supplementary material available at 10.1007/s00294-025-01325-w.

## Introduction

The tips of eukaryotic linear chromosomes consist of special nucleoprotein structures known as telomeres. Telomeres protect the chromosome ends from degradation and fusion with other chromosomes. Complete DNA replication at the chromosome ends is not possible by the canonical machinery, leading to chromosome shortening with each cell division, a process known as the end replication problem. If cells continue to proliferate, telomere shortening eventually triggers an irreversible cell cycle arrest called replicative senescence. It has been reported that five dysfunctional telomeres are required to activate senescence in human cells, while only one dysfunctional telomere is enough to trigger senescence in telomerase-deficient budding yeast (Abdallah et al. 2009; Kaul et al. 2011). To acquire replicative immortality, cancer cells must prevent the telomere shortening by expressing telomerase, a ribonucleoprotein complex specialized in adding short repeats to the telomeres by copying its built-in RNA template (Autexier et al. 1996). Telomerase reactivation is a prerequisite step for most tumor immortalization, but 10–15% of tumor types acquire alternative pathways to maintain their telomere length for proliferation, collectively known as Alternative Lengthening of Telomeres (ALT) (Bryan et al. 1997; Pickett and Reddel 2015). Telomerase is an attractive target to treat cancer, but given that ALT plays a vital role in protecting telomere and genomic integrity in cancer cells, therapeutic approaches targeting the ALT pathway are also being developed (Lu et al. 2019).

Yeast has constitutively active telomerase and has proven to be a useful model to study the molecular processes that occur in the absence of telomerase (Cohn and Blackburn 1995). Similar to mammalians, yeast also acquires recombination-based alternative pathways to maintain telomere length following telomerase inactivation (Lundblad and Blackburn 1993; McEachern and Blackburn 1996; Lundblad 2002; Kockler et al. 2021). When telomerase is inactivated in *Saccharomyces cerevisiae* cells, they divide for a finite number of generations. Like human somatic cells, they experience gradual telomere shortening and eventually undergo replicative senescence. Only a small subpopulation of the cells escapes the severe growth crisis, and those survivors maintain their telomeres by activating recombination-mediated ALT pathways, which act via homology-directed DNA repair and break-induced replication (BIR) processes. Two different kinds of survivors have been identified, characterized by their use of either subtelomeric (type I) or telomeric (type II) sequences to maintain telomeres and genome stability (Chen et al. 2001). Both types show genetic dependence on *RAD52* for viability, but differences in other genetic factors. While type I survivors depend on *RAD51* to amplify subtelomeric Y’ elements, type II cells depend on *RAD59* and *MRX* function and have elongated telomere repeats (Chen et al. 2001). However, recent studies suggest that these pathways may not be entirely independent, and that the factors may act together in a unified ALT pathway (Kockler et al. 2021).

The TG-rich telomere sequences associate with specialized DNA-binding proteins to form a chromatin cap that protects the chromosome ends from detection by DNA damage repair proteins and from chromosome end-to-end fusions. In *S. cerevisiae*, the telomere binding protein Rap1 binds to the double-stranded part of the telomeric sequence, while Cdc13 binds the single-stranded 3’ overhang (Conrad et al. 1990; Nugent et al. 1996). Recruitment of other proteins leads to the formation of a specialized telomere chromatin, which can form a foldback structure, a dynamic type of interaction that caps the end to shield away the 3’ overhang from exonucleolytic attacks, and is postulated to regulate the access of telomerase (Pryde and Louis 1999; Poschke et al. 2012; Pasquier and Wellinger 2020). In addition to its role in telomere protection, Rap1 is involved in transcriptional regulation of several genes. Although it performs a highly sequence-specific binding to DNA, it exhibits a capability of flexible binding to different promoter sequences as well as telomeric sequence variants (Wahlin and Cohn 2000, 2002). In senescing *S. cerevisiae* cells, the shortened telomeres lead to relocalization of Rap1 to promoter regions of new target genes, having an impact on overall gene expression levels and the rate of senescence (Platt et al. 2013). Rap1 was shown to bind with a lower affinity to these new binding sites, which could be explained by their lack of the previously defined Rap1 consensus sequence.

Subtelomeres are defined as the genomic regions immediately adjacent to the telomeres. The size of the subtelomeric regions vary and are hard to estimate since the internal boundary usually cannot be clearly defined. Subtelomeres are rich in repetitive sequences and therefore show an increased rate of recombination, rendering them highly variable and dynamic genomic regions with a high divergence rate (Cohn et al. 2006; Snoek et al. 2014; Garcia-Rios et al. 2017). These features form a basis for generation of diversity and invention of novel genes, and bring possibilities to adapt to new environments. Telomeres are known to impose chromatin-mediated silencing on genes located in the vicinity, known as the telomere position effect (TPE), and subtelomeres could serve to block the spreading of heterochromatin to the genes located more internally (Tashiro et al. 2017). Moreover, subtelomeres may influence the telomere length in cis, and it was shown that the subtelomeric proteins Tbf1 and Reb1 act as negative regulators of telomere length in *S. cerevisiae* (Berthiau et al. 2006). Tbf1 and Reb1 bind to the subtelomeric repeat elements X and Y’ that are present just proximal to the telomeric sequences. The X elements are variable in size (300 bp – 3 kb) and present at every telomere, while only 17 of the 32 chromosome ends harbor the quite well conserved Y’ elements that exist in two major size classes (6.7 kb and 5.2 kb) (Louis and Haber 1992). Therefore, the subtelomere-telomere junction differs in between the chromosome ends; some ends contain the Y’-element right at the junction, whereas some ends contain the more variable X-element (Cohn et al. 2006; Snoek et al. 2014). Despite this heterogeneity, the overall in vivo chromatin organization was observed to be similar for all telomeres, with Tbf1 and Reb1 interacting with a nucleosome-free subtelomeric region just adjacent to the telomere (Pasquier and Wellinger 2020). Studies in *S. cerevisiae* show that subtelomeres play important roles for the survival of cells after loss of telomerase (Jolivet et al. 2019). In the type I survivors the telomeres are stabilized by amplification and dispersal of Y’ elements, generating long tandem arrays (Lundblad and Blackburn 1993; McEachern and Blackburn 1996; Lundblad 2002; Kockler et al. 2021). Strikingly, in strains constructed to harbor Y’ elements on all telomeres, the cells did not experience a replicative senescence crisis and only type I survivors were observed after telomerase loss, implying that the selection of recombination pathway might be influenced by the nature of the sequences present in the subtelomeres (Grandin et al. 2020). Also in the fission yeast *Schizosaccharomyces pombe*, amplification of subtelomeric elements was shown to stabilize linear chromosomes in rare telomerase-deficient survivors, although chromosome circularization is the most prevalent rescue mechanism (Apte et al. 2021). Thus, a substantial amount of data supports the notion that subtelomeres play important roles in wild-type cells and provide backup mechanisms for telomere maintenance and continued proliferation in telomerase-deficient cells.

The budding yeast *Naumovozyma castellii* belongs to the family *Saccharomycetaceae* and has been extensively used in comparative genomics, where it was shown that a whole-genome duplication followed by massive gene loss has resulted in a genome size of 11.2 Mb distributed across ten chromosomes (Karademir Andersson and Cohn 2017). Like most eukaryotes, *N. castellii* telomeres consist of short and regular telomeric repeats, TCTGGGTG, which has facilitated the identification of binding sites for the telomere proteins Rap1 and Cdc13 and the characterization of the initiation of telomerase extension (Wahlin and Cohn 2002; Rhodin et al. 2006; Karademir Andersson et al. 2017). Rap1 requires sequence-specific interaction with a 12 bp minimal binding site (MBS) (5′-GGGTGTCTGGGT-3′) on the double-stranded region, while the single-stranded 3’ overhang is bound by Cdc13 using an eight nucleotide MBS (5′-GTGTCTGG-3′). These two proteins exhibit complementary roles in preventing exonucleolytic degradation of telomeric ends, where notably, Rap1 can protect the single-stranded 3’ overhang when bound across the ds-ss junction (Runnberg et al. 2017, 2019; Itriago et al. 2022). These results align with the function of *S. cerevisiae* Rap1 to prevent the accumulation of single-stranded DNA in both dividing and non-dividing cells, and it was suggested that protection of the DNA ends may be provided by distinct configurations of the telomeric cap during different phases of the cell cycle (Vodenicharov et al. 2010).

We previously showed that telomerase-deficient *N. castellii* cells experience rapid progressive telomere loss after disruption of telomerase activity (Cohn et al. 2019). Strikingly, *N. castellii* effectively switches to the telomerase-independent ALT pathway without exhibiting any pronounced senescence phenotype, showing only a slight reduction in growth rate (Cohn et al. 2019). The *N. castellii* ALT pathway is dependent on the recombination genes *RAD52* and *RAD51*, but does not require *RAD59* or *RAD50* (Itriago and Cohn, manuscript in prep.). Hence, regarding the genetic requirements, it resembles the type I survivor pathway identified in *S. cerevisiae* (Chen et al. 2001). Terminal Restriction Fragment (TRF) assays of *N. castellii* ALT strains showed that short and distinct telomere structures establish early and then persists during extensive serial re-streaking procedures, implying a stabilization of the end structure at a concise length. Examination of the terminal sequences revealed the presence of the same telomere-adjacent subtelomeric structure as in the wild-type (WT) strain, with the only difference being the shortened ∼50 bp telomeric sequences, compared to the WT ∼320 bp (Cohn et al. 2019). However, ALT strains occasionally exhibit extension of their telomeres, appearing in the TRF assay as a strikingly distinct ladder pattern with uniform increments. The ladder pattern is formed by arrays of uniform repeats containing the most telomere-adjacent subtelomeric region, termed TelKO element, and flanking telomeric sequences. This implies that the TelKO element is used as a copy template in the telomere extension process.

In this study, we aimed to analyze the telomere structures in greater detail, and to determine the dynamics and frequencies of short telomere structures versus extreme elongation events. We also asked whether the terminal structures show any sequence variations, which would be useful in elucidating whether several different telomeres are used as template for the elongation events. Indeed, we identified two main different length variants of the TelKO element, where the short variant (220 bp) is a truncated version of the long (445 bp) variant. Strikingly, in each line of ALT cells, a specific variant effectively spreads to all chromosome ends and PacBio whole genome sequencing of an ALT strain showed the presence of a uniform novel structure at all subtelomeres. Thus, even though the wild-type strain contains both elements, only one of them remains in the respective ALT strain, resulting in a homogenization of the subtelomeric sequences. We show that the telomeric protein Rap1 can bind to the TelKO element, suggesting that short telomeres may be stabilized by inward expansion of the telomere chromatin to include the subtelomeric region. Hence, the subtelomeric TelKO element is the key for the effective establishment of ALT cells and provides the solution for sustained long-term growth.

## Materials and methods

### Strains and media

The yeast *Naumovozyma castellii* was previously called *Saccharomyces castellii* or *Naumovia castellii*. The *N. castellii* strains used in this study were: Y235 (*MATa/MATα*,* ura3/ura3*), YMC133 (*MATa/MATα*,* ura3/ura3*,* tlc1::kanMX3/tlc1::kanMX3*), YMC48 (*MATα*,* hoΔ::hphMX4*,* ura3*), YMC481 and YMC482 (*MATα*,* hoΔ::hphMX4*,* ura3*,* tlc1::kanMX3*) (Astromskas and Cohn 2007, 2009; Karademir Andersson et al. 2016). The *TLC1* gene was disrupted by using the TLC1-kanMX3 construct, with the kanMX3 cassette inserted into the HpaI site of the *N. castellii TLC1* gene, as previously described (Astromskas and Cohn 2009). The Y235 strain has a functional *HO* gene, leading to mating and production of diploid homozygous strains after the isolation of haploid knockout spores. All yeast strains were grown at 25 °C in YPD medium containing 1% (w/v) yeast extract, 2% (w/v) peptone and 2% (w/v) glucose, and plates contained 2% (w/v) agar.

## Serial streaking assay

After sporulation and microdissection of asci, tetrad spores were germinated and grown into colonies at 25 °C for 48 h (Streak 1; S1). Colonies were then re-streaked onto fresh YPD plates (Streak 2; S2). After 48 h incubation at 25 °C, single colonies were picked and re-streaked again (Streak 3; S3). Re-streaking was repeated to obtain up to 16 subsequent streaks (S1-S16), where each passage corresponds to 20–25 generations.

## Genomic DNA isolation

A single yeast colony was used to inoculate 10 ml of YPD medium and grown overnight at 25 °C, 200 rpm. Next day, 2 ml of the overnight culture were used to inoculate 50 ml of YPD medium. The cell density was measured with a Nucleocounter^®^ YC-100™ (Chemometec) and the culture was incubated at 25 °C and 200 rpm until a cell density of 5 × 10^7^ cells/ml was reached. Genomic DNA was extracted using the Wizard^Ⓡ^ Genomic DNA purification kit (Promega), with some modifications of the manufacturers protocol. To digest the yeast cell wall, the pelleted cells were incubated with 5 U/ml Zymolyase 20T (US Biological) at 37 °C for 40 min. The isolated genomic DNA was dissolved in 50 mM Tris-HCl (pH 8.0) and the DNA concentration was quantified with a Qubit^Ⓡ^ 3.0 Fluorometer (Invitrogen).

Genomic DNA for the whole genome sequencing was isolated with the Genomic-tip 500/G (Qiagen), following the protocol from the manufacturer. Yeast cultures were grown in YPD to a cell density of 3 × 10^8^ cells/ml. For each column, 2 × 10^9^ cells were pelleted and then incubated with 83 U/ml Zymolyase 20T (US Biological) at 30 °C for 30 min, followed by treatment with 0.53 mg/ml Proteinase K at 50 °C for 30 min. The purified DNA was finally resuspended in 500 µl 10 mM Tris-HCl (pH 8.0).

## Terminal restriction fragment assay (TRF assay)

Telomere length was analyzed by TRF assay, using 300 ng of *N. castellii* genomic DNA digested with HinfI (Thermo Scientific). The digested DNA was resolved on a 0.8% agarose gel in 0.5 x TBE buffer, and was blotted to a nylon membrane (Hybond-XL, Amersham or Biodyne B, Pall). The dried membrane was crosslinked by exposure to UV light at 120 mJ/cm^2^. Hybridization probes were 5’end-labeled by T4 polynucleotide kinase and [γ^32^P]-ATP. The membrane was hybridized with a labeled probe overnight and then washed once for 5 min at RT and then twice for 15 min in 100 mM Na_2_HPO_4_ and 2% SDS at probe-specific temperatures. Oligonucleotides used: telomeric probe (TGTCTGGG)_2_ hybridization at 40 °C and wash at 45 °C, universal subtelomeric probe (GTTGGAGGTATTTGTTGTTGATG) hybridization at 45 °C and wash at 50 °C, TelKO_445_-specific probe (GGAATATGGTGGGATGGAATAGGTAG) hybridization at 45 °C and wash at 55 °C. Typhoon FLA 9500 (GE Life Sciences) was used to visualize the signals on the membrane.

## BAL-31 assay

Genomic DNA was incubated with 0.25 U/µg gDNA of BAL-31 nuclease (NEB) for increasing amount of time at 30 °C. At each time point, an aliquot of the reaction was withdrawn, and the enzyme was heat-inactivated at 65 °C for 10 min in the presence of 33 mM EGTA and stored on ice. All aliquots were then cleaved with HinfI and the DNA was separated on a 0.8% agarose gel (270 ng DNA per well) and subjected to Southern blot analysis as described above.

### Telomere-PCR

To obtain the sequence present in the DNA terminus we used Telomere-PCR, where the genomic DNA 3’ends were C-tailed using dCTP and Terminal Transferase, followed by PCR amplification. To C-tail the genomic DNA (gDNA), 200 ng gDNA was denatured in 20 mM Tris-acetate, 50 mM potassium acetate, 0.25 mM cobalt chloride, 20 mM magnesium acetate, and 0.1 mM dCTP at 98°C for 10 min and tailed with 0.2 U Terminal Transferase (NEB) at 37°C for 30 min, followed by heat-denaturation at 70°C for 10 min. For non-tailed DNA, the enzyme was replaced by H_2_O. For the PCR amplification, 8 ng C-tailed or non-tailed gDNA was put in a 50 µl reaction mix of 200 µM of each dNTP, 0.6 µM forward primer (Subtel-F3), 0.6 µM reverse primer (poly(G)_18_), 1x HF buffer and 0.02 U/µl Phusion High-Fidelity DNA Polymerase (Thermo Scientific). The PCR program consisted of initial melting at 98° for 2 min, followed by 45 cycles of 30 s denaturation at 95°C, 30 s annealing at 64°C, 1 min extension at 72°C, and a final elongation step of 5 min at 72°C. Amplification products were analyzed by electrophoresis on 0.8% agarose gel. The correct size bands were gel extracted and sequenced (Sanger sequencing, Eurofins). SnapGene was used for alignment of DNA sequences. Primers used in Telomere-PCR: Subtel-F3, 5’-TGGTTGTAATATTGGAATTATTTTGTAATTCTAGTTCCCCGTTGGG-3’; poly(G)18, 5’- TGCTCCATACATTACTTAT(G)_18_ -3’.

## Whole genome DNA sequencing and bioinformatic analyses

PacBio DNA sequencing was performed at the NGI-UGC facility (SciLife Lab, Uppsala, Sweden). The raw sequencing reads were processed using the software Canu for de novo chromosome contig assembly, followed by consensus polishing by Medaka (ONT). The software TeloClip was used to identify DNA ends containing telomeric sequences from the raw reads (https://github.com/Adamtaranto/teloclip) to extend the consensus contigs.

## Electrophoretic mobility shift assay (EMSA)

The recombinant *N. castellii* Rap1 full-length protein was expressed in *E. coli* BL21 cells and extracts prepared as previously described (Wahlin and Cohn 2000, 2002). Double-stranded (ds) oligonucleotides were prepared by annealing equimolar amounts of the respective forward and reverse strands in 1 mM Tris-HCl, pH 8.0, 0.1 mM MgCl_2_, by boiling for 2 min and slowly cooling down to room temperature. A ds-oligonucleotide with a wild-type telomeric sequence was used as the labeled probe: 5’-GTCTGGGTGTCTGGGTGTC-3’ (19 bp). The 5’ ends of the telomeric ds-probe were radioactively labeled with [γ^32^P]-ATP using T4 polynucleotide kinase (New England Biolabs), purified on Illustra Microspin G-25 columns (GE) and diluted in 10 mM Tris-HCl, pH 7.5. In 15 µl binding reactions, 10 fmol labeled probe was mixed with 1.5 µg non-specific competitor mix (0.5 µg each of sheared *E.coli* DNA (∼250 bp), salmon sperm DNA and yeast t-RNA) and ∼0.6 µg Rap1 protein extract in 1x Binding buffer (10 mM Tris-HCl, pH 7.5, 7 mM MgCl_2_, 8% glycerol). To compare the binding affinity to different sequences, unlabeled competitor oligonucleotides were added into the reaction in molar excess of the probe prior to protein addition; telomeric sequence in 40x, 400x and subtelomeric sequences (ST-1-ST-4, Fig. 7) in 400x, 4000x molar excess. The binding reaction was incubated at 25 °C for 15 min and then loaded onto a non-denaturing 5% polyacrylamide gel (29:1 acrylamide: bis-acrylamide) and run in 1x TBE (89 mM Tris-borate, 2 mM EDTA, pH 8.0), 150 V at 4 °C. Radioactive signals from dried gels were visualized with a Typhoon FLA 9500 biomolecular imager (GE Life Sciences). Signals in the respective lanes were quantified with the ImageQuant TL software. Bands were automatically detected with the settings: minimum slope 15, median filter 10, % maximum peak 0. The fraction (%) of bound probe was calculated in each lane as (shifted signal)/ (total signal of shifted and unshifted bands). The fold change of the ST-1-4 compared to the telomeric sequence was calculated as the ratio (ST: telomeric sequence) and reported as the mean value of five technical repeats with the standard deviation.

## Results

### *N. castellii* ALT cells maintain shortened telomeres throughout extensive passaging

**Fig. 1:**
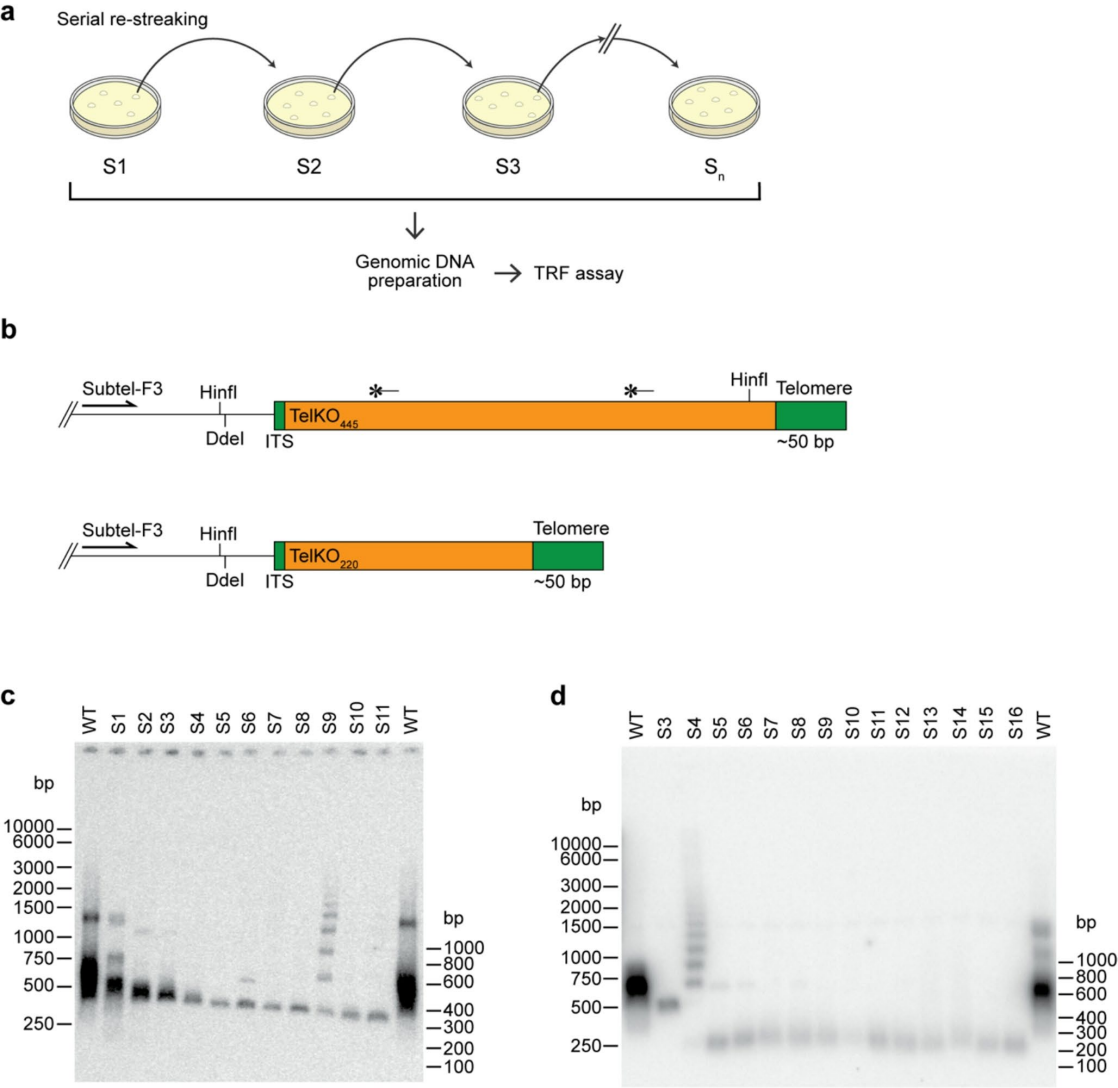
Short telomeres are stably maintained in *N. castellii* ALT cells. (**a**) Experimental overview of the serial streaking assay of yeast colonies and telomere length analysis. After spore germination on the S1 agar plate, colonies were successively re-streaked, S2-S_n_. (**b**) Schematic overview of the subtelomeric regions in *N. castellii* containing two variants of the TelKO element; TelKO_445_ and TelKO_220_, respectively. In the WT strains the subtelomeric TelKO element is located just internally of the ∼320 bp telomeric sequence in a subset of the 20 chromosome ends. In the ALT strains, the telomeric sequence is shortened to ∼50 bp. The locations of the two subtelomeric hybridization probes are indicated with black bars above the TelKO_445_ element, the left one is a universal probe and the right one is specific for TelKO_445_. Subtel-F3, forward primer used in the Telomere-PCR. (**c, d**) Terminal restriction fragment (TRF) assay of telomerase-deficient strains in serial re-streaking procedures. (**c**) Haploid wild-type (WT) parental strain (YMC48) and ALT strain (YMC481, *tlc1*^*−*^), streaks S1-S11. (**d**) Diploid WT parental strain (Y235) and ALT strain (YMC133, *tlc1*^*−*^*/ tlc1*^*−*^), streaks S3-S16. Genomic DNA was digested with HinfI, separated on a 0.8% agarose gel and analyzed by a Southern blot hybridization with a 16-mer telomeric sequence probe

*N. castellii* telomerase knockout cells effectively activate the Alternative Lengthening of Telomeres (ALT) pathway to maintain telomere homeostasis via recombination, which provides the solution for sustained long-term growth (Cohn et al. 2019). To investigate the maintenance of telomeres in ALT cells, we disrupted the telomerase RNA gene (*tlc1*^−^) and performed a serial streaking assay (Fig. 1a) (Cohn et al. 2019). Telomere length and structure was analyzed in a terminal restriction fragment (TRF) assay, cleaving the genomic DNA with the restriction enzyme HinfI (Fig. 1c-d). In the *N. castellii* wild-type (WT) strain, the main part of the terminal DNA fragments exhibits a smear with a mean length of ∼600 bp when hybridized with a telomeric sequence probe (Fig. 1c). During passaging of the telomerase knockout cells, the telomere smear is progressively decreasing in size within the first streaks, until reaching a size of ∼350 bp. The shortened telomeres in the ALT cells show a narrower and more distinct band in the TRF assay, compared to the WT smear. This short and distinct band would generally form at streak 3–4 (S3-S4) corresponding to ca. 60–80 generations from the telomerase disruption (∼20 generations per streak). Remarkably, we observed that the distinct ∼350 bp telomeric band is retained in the continued serial streaking of the ALT colonies, indicating maintenance of a short and stable structure. Although this is a general feature, occasional telomere lengthening events occur, as visualized by the distinct ladder pattern of bands with regular increments (Fig. 1c, S9). These elongations are due to stochastic additions of long arrays of repeats consisting of the subtelomeric TelKO element and telomeric sequences, characteristic of the ALT recombinational telomere elongation events in *N. castellii* (Cohn et al. 2019).

Intriguingly, the ladder patterns sometimes appear only in a few separate streaks of the assay, leaving no trace in the subsequent passaging. Thus, several of the streaking assays show only the shortened structure, where the telomeres are presumably stabilized without any addition of repeat arrays, seemingly containing just the wild-type subtelomeric sequence and a residual ∼50 bp telomeric sequence (Cohn et al. 2019). This rather contradictory finding piqued our interest to further investigate the ALT telomeres in greater detail to determine the dynamics of their structure.

### Different TelKO element variants can stabilize and maintain the ALT telomere structure

Interestingly, in some of the serial streaking assays of the telomerase-deficient strains, we discovered a different TRF band pattern, where the telomeric bands were drastically decreased in size to ∼200 bp and showed a much lower hybridization signal (Fig. 1d, S5-S16). Notably, once the new band profile appeared in a streak, it was retained in the following passages, hence indicating that the chromosome end is stabilized by a terminal structure different from the previously characterized TelKO element.

To decipher the terminal DNA sequence present in the different ALT cell lines, we performed Telomere-PCR, followed by gel extraction and sequencing of the amplicon bands. Genomic DNA was C-tailed, and Telomere-PCR performed using a forward primer directed towards the subtelomeric region internally of the TelKO element and a reverse primer annealing to the C-tail. In this way, we recovered two different variants of the TelKO element; a longer 445 bp variant (TelKO_445_) and a shorter 220 bp variant (TelKO_220_), which is a truncated version of the longer one (Fig. 1b). The short TelKO_220_ variant recovered here is identical in sequence to the previously described element (Cohn et al. 2019). The newly discovered TelKO_445_ element variant contains a 225 bp extension and a HinfI recognition site near its terminus (Suppl. Fig. S1a), which explains the very short telomeric band observed in the TRF assay (Fig. 1d, S5-S16). Consistent with this, re-hybridization of the membrane in Fig. 1d with a subtelomeric probe revealed a band corresponding to the internal HinfI fragment, thus verifying the structure of the TelKO_445_ element (Suppl. Fig. S1b).

Thus, we have unveiled two variants of the subtelomeric TelKO element, identical in their overlapping regions, which are both able to maintain ALT telomeres. Interestingly, the serial streaking in Fig. 1d displays a marked and clear shift of the TRF band pattern, from the ∼350 bp to the ∼200 bp telomeric band (Fig. 1d, S3 and S5), indicating that the TelKO_220_ variant is replaced by the TelKO_445_ variant during the passaging. Notably, the streak in between these samples shows a ladder pattern (Fig. 1d, S4). This makes it tempting to speculate that telomere shortening in the founder cell of this S4 population triggered widespread telomere lengthening activities, eventually leading to the recombinational replacement of TelKO elements in most telomeres in S5.

### The TelKO elements are located at the chromosome termini

**Fig. 2:**
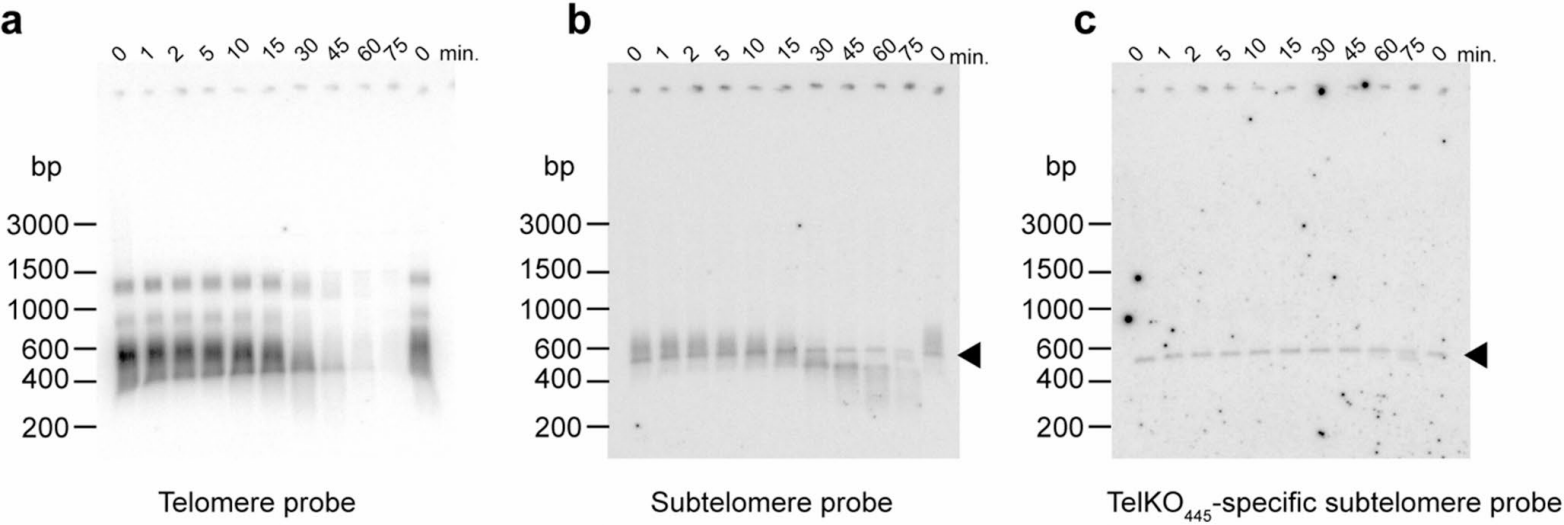
BAL-31 assays show the presence of the two variants of the TelKO element at the DNA ends. Genomic DNA of the WT strain (YMC48) was cleaved with BAL-31 for increasing amount of time (0–75 min as indicated at the top), followed by the TRF assay procedure using HinfI digestion. The membrane was first hybridized with the telomeric sequence and subsequently re-hybridized twice with different subtelomeric sequences as hybridization probes (probe indicated under the respective image); (**a**) telomeric probe, (**b**) universal subtelomere probe; a sequence shared by both TelKO element variants, (**c**) TelKO_445_-specific probe; a sequence specific for the TelKO_445_ variant. See Fig. 1 for the positions of the respective subtelomere probes

To verify that the TelKO elements are terminally located, we performed a BAL-31 assay. The BAL-31 exonuclease digests the genomic DNA from the ends and inwards. Wild-type genomic DNA (YMC48) was treated with the exonuclease BAL-31 for increasing amounts of time, and then the time point samples were cleaved with HinfI to release the terminal fragments, followed by separation on an agarose gel and Southern blot transfer. For the hybridization, we firstly used the telomeric probe, which gives the typical smeary telomeric ∼600 bp signal in the sample at time 0 which is undigested by BAL-31 (Fig. 2a). With increasing BAL-31 digestion time, the band is gradually decreasing in size until finally almost totally disappearing at 60 min. This quick and synchronized disappearance of the total signal shows that the telomeric sequences are residing in the termini of all chromosomes, as previously demonstrated for *N. castellii* (Cohn et al. 2019).

Next, to investigate the subtelomeric sequences, we stripped and re-hybridized the membrane with a probe targeting the common region of the TelKO elements; universal probe (Fig. 2b). In the undigested sample (time 0), the subtelomeric probe produces a narrower signal which is overlapping with the telomeric signal retrieved in the first hybridization. The bands decrease with a similar time course profile as seen with the telomeric sequences, thus confirming the terminal location of the TelKO elements. However, this probe revealed two bands with different behaviors in the time course. While the smeary upper band shows a marked decrease in size already from 30 min, the lower more distinct band (indicated by arrowhead) is not affected until at 75 min. This result presumably depends on the presence of different TelKO variants in the respective bands. The TelKO_220_ element is fully located within the terminal HinfI fragment and is therefore predicted to show a smeary signal that is immediately affected by the BAL-31 enzyme. In contrast, the additional HinfI site in the TelKO_445_ element produces an internal ∼470 bp band, which is unaffected until BAL-31 digestion extends beyond this distal HinfI site. To validate this notion, we re-hybridized the membrane with a probe specific for the TelKO_445_ element (Fig. 2c). As expected, this probe detected only the narrow lower band observed with the universal subtelomere probe, which remained unaffected until the final time point. In conclusion, the BAL-31 results confirm the presence and terminal location of the different TelKO element variants on the chromosomes. To investigate whether the TelKO elements remain terminally located in the ALT cells we performed the BAL-31 assay on a strain having the characteristic short telomere structure (Suppl. Fig. S2). The telomeric sequence hybridization signal showed a similar disappearance in ALT and WT samples (Fig. 2, Suppl. Fig. S2a). Furthermore, re-hybridization of the membrane with the universal subtelomere probe shows that the BAL-31 cleavage affects the TelKO sequences with just a minor delay in the time points (Suppl. Fig. S2b). This result shows that the TelKO elements remain terminally localized on the chromosomes in the ALT cells.

### Screening populations of colonies reveals a stable and homogeneous telomere structure

**Fig. 3:**
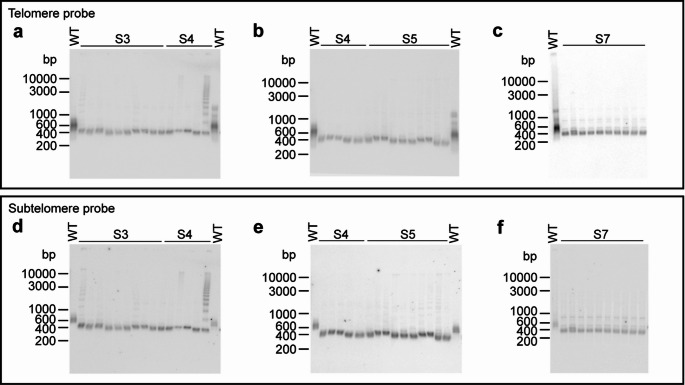
Screening of large populations of colonies indicate a stable and homogeneous telomere structure in the ALT cells. TRF assays were performed on ten separate colonies isolated from each of the indicated streaks (S3, S4, S5, S7) in the serial streaking procedure of an ALT strain (YMC133, *tlc1*^*−*^*/ tlc1*^*−*^). The membranes were first hybridized with a telomere probe (**a-c**) and then re-hybridized with a subtelomere probe detecting the TelKO element (**d-f**)

To investigate the dynamics of the ALT telomeres, we performed TRF assays on several independent colonies of specific streaks. Analysis of 10 independent colonies from a diploid ALT strain (*tlc1*^*−*^/*tlc1*^*−*^) from streak 3 (S3), showed that they all exhibit a distinct and prominent telomeric band signal at ∼350 bp, characteristic of the presence of the subtelomeric TelKO_220_ element (Fig. 3a). This result indicates that the terminal structure established in the ALT cells of the parental colony is stably kept in the population of cells that grow into the S3 colonies. The conformity of the bands and the absence of major deviations indicate that an invariant shortened telomere structure is effectively stabilizing the telomeres. Furthermore, for the successive streaks of this strain the same result was obtained, with the predominant telomere signal appearing in the same distinct ∼350 bp band for colonies from S4, S5, S7 and S10, (Fig. 3b-c, Suppl. Fig. S3a). A similar result was obtained for a haploid ALT strain (*tlc1*^*−*^), which gave the same ∼350 bp band in 10 separate colonies of the streaks S3, S7 and S13 (Suppl. Fig. S3b-c). To investigate the subtelomere structure, we stripped and re-hybridized the TRF membranes with the universal subtelomeric TelKO probe, which revealed the expected distinct band, indicating a homogeneous structure (Fig. 3d-f).

The characteristic ALT ladder pattern appears in some of the samples, thus suggesting telomere lengthening with the TelKO element. In both the diploid and haploid strains, only sporadic ladder patterns were observed, exhibiting a similar frequency. Thus, we can conclude that diploid and haploid strains show a conformity regarding the maintenance of ALT telomere structures. Notably, after longer exposures of the TRF membranes, a very faint ladder pattern can sometimes be discerned in some of the other samples (Suppl. Fig. S3d). This implies that array lengthening is occurring at a low frequency, in a small number of cells in the liquid culture.

In the same way, we performed a population screen of 10 colonies from each of two different streaks from which we discovered the new TelKO_445_ variant. Consistent with the results above, all analyzed colonies within these streaks showed an identical band pattern, hence indicating a stable telomere structure also in telomeres containing the TelKO_445_ variant (Suppl. Fig. S3e). To verify the subtelomeric structure, we re-hybridized with the universal subtelomeric TelKO probe, which showed a homogeneous structure with the expected internal HinfI band in all samples (Suppl. Fig. S3f). Subsequent rehybridization of the membrane with a probe specific for TelKO_445_ showed a comparable signal to the universal probe, verifying that the ALT cells in all these colonies indeed contain an abundant amount of this TelKO variant (Suppl. Fig. S3g).

Taken together, our results show the presence of a stable shortened telomere structure in the ALT cells. Notably, the short telomere structure that is established in the early stages of the passaging is stably kept throughout extensive numbers of successive streaks and is the predominant structure found in the population of cells. The stochastic appearance of the array ladder pattern, and the fact that ladder pattern samples also retain the short ∼350 bp band, indicates that the dramatic elongation with the TelKO element is a rather rare event that is not taking place in all cells in a population, and it may not take place in all telomeres within the cell.

### The TelKO elements exhibit a homogeneous sequence in the ALT strains

**Fig. 4:**
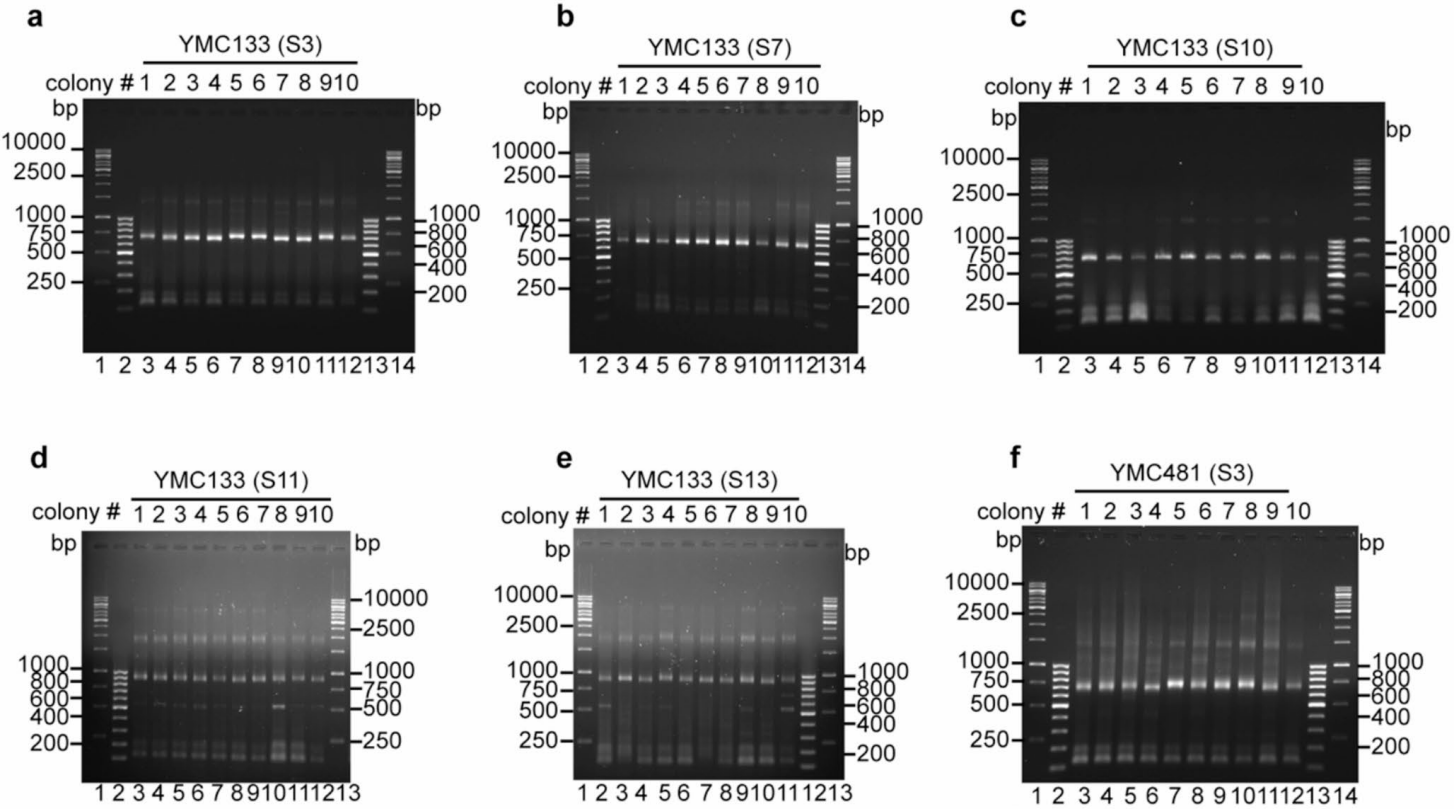
ALT cells maintain a homogeneous terminal structure within the respective streak population. Telomere-PCR analysis was performed on genomic DNA from ten colonies of the respective streak. (**a-c**,** f**) An amplicon band of ∼700 bp, indicative of the TelKO_220_ variant, is generated in ALT strains YMC133 (*tlc1*^*−*^*/ tlc1*^*−*^) streaking assay #1 (S3, S7, S10) and YMC481 (S3). (**d-e**) An amplicon band of ∼900 bp, indicative of the TelKO_445_ variant, is generated in streaking assay #2 of YMC133

We further wanted to analyze the terminal DNA sequences of the isolated colonies from the population screen. To this end, we performed Telomere-PCR on the genomic DNA from the 10 separate colonies of the respective streaks. As expected, the colonies within each streak showed a conformity, generating the same size of the PCR amplicon band (Fig. 4, Suppl. Fig. S4). ALT strains containing the TelKO_445_ element versus the TelKO_220_ element generate a ∼900 bp and ∼700 bp amplicon band, respectively. Amplicons were extracted and sequenced from three colonies of each streak. Sequence analysis revealed identical TelKO elements among colonies within each respective streak (Cohn et al. 2019). Moreover, both the TelKO_445_ and TelKO_220_ elements show identical sequences even when isolated from separate streaks (in total 10 streaks analyzed). Thus, we conclude that the TelKO elements are kept highly homogeneous throughout extensive numbers of generations in the serial streaking.

This result is further underlined by re-hybridization of the TRF assay membranes with a probe specific for the TelKO_445_ element, detecting the second extended half of the element. Samples containing the TelKO_445_ element showed very strong and prominent signals in all samples from the streak, indicating an amplification of the TelKO_445_ elements (Suppl. Fig. S3g, S13). In contrast, colonies from a streak from a different serial line known to contain the TelKO_220_ element showed a weaker signal than WT, thus indicating a reduction of TelKO_445_ elements and confirming that the TelKO_220_ element is predominantly present in these samples (Suppl. Fig. S3g, S7). This remarkable result is indicative of an effective spreading of a specific element in the respective ALT strains, leading to homogenization of the sequences in all subtelomeres.

### Subtelomeres are undergoing sequence homogenization in the establishment of ALT cells

To get more insight into the subtelomere structure in different chromosome ends, we performed PacBio whole genome sequencing of a WT *N. castellii* strain (YMC48) and assembled chromosome contigs using TeloClip and Canu. We could locate telomeric sequences and retrieve the adjacent subtelomeric regions in 18 of the 20 chromosome ends. Consistent with the BAL-31 results, we confirmed that TelKO elements are exclusively located in the subtelomeres, present as single copies just internal to the telomeric sequence repeats. Hence, the two variants, TelKO_445_ and TelKO_220_, are localized to separate chromosome ends. Moreover, as previously observed in TRF assays, some chromosome ends are lacking TelKO elements (7/18), and some contain partial TelKO_445_ elements that are extending beyond TelKO_220_ to various extent (Cohn et al. 2019). Thus, regarding the TelKO elements, the subtelomeres show a quite variable content in the WT strain.

In the same way, we performed PacBio sequencing of an established ALT strain (YMC481, S5). Remarkably, the sequence data shows that all the chromosome ends in this ALT strain have acquired the same TelKO variant; the short TelKO_220_, thus confirming the subtelomere homogenization observed in the above TRF assays. This means that even telomeres lacking a TelKO element in the WT strain acquired one in the ALT strain. Moreover, it means that a TelKO variant switch has occurred, where the TelKO_445_ variant present in the WT telomere was replaced with the TelKO_220_ variant in the ALT strain. Detailed analysis further revealed that the WT strain contains two different alleles of the TelKO_220_ sequence, differing by a single nucleotide at position 28 (T or C) (Suppl. Fig. S1a). Remarkably, only one of these alleles (C) is present in all telomeres of the ALT strain. Hence, together these observations further substantiate the homogenization of telomeres in the ALT strains.

**Fig. 5:**
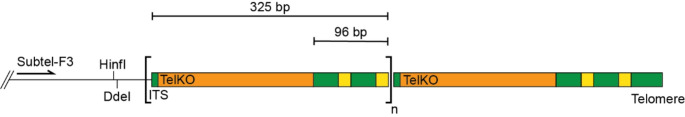
The subtelomeres of ALT cell chromosomes contain arrays of composite higher-order repeats. Schematic overview of the repeated unit found in arrays at all chromosome ends of the sequenced ALT strain (YMC481). The 325 bp higher-order repeat contains the TelKO_220_ variant, flanked internally by a short 9 bp interstitial telomeric sequence (ITS, green box) and distally by a 96 bp element containing two identical 35 bp segments of telomeric sequence (green) and two 13 bp segments identical to the end of the TelKO element (yellow). The number of higher-order repeats vary between 2–27 (denoted by n). The sequence at the extreme terminus has the same structure, followed by a terminal telomeric sequence that is shorter than in WT cells

In the contig assembly of the ALT strain, we observed long elongated arrays of TelKO elements in some of the telomeres, which is correlating to the ladder pattern observed in the TRF assays. Notably, the TelKO_220_ elements in these arrays are all the same allele (C). A closer analysis of the arrays revealed a 325 bp repeated unit with a composite structure, where the TelKO_220_ element is flanked by telomeric sequences and additional 13 bp partial TelKO segments that are identical to the end of the TelKO_220_ element (Fig. 5, Suppl. Fig. S5). Thus, the 325 bp unit forms a higher-order repeat, containing smaller repeated units. The 13 bp terminus of TelKO_220_ is present in triplicates within this higher-order repeat, separated by identical copies of a 35 bp telomeric sequence stretch (Fig. 5, Suppl. Fig. S5). It is noteworthy, that this 325 bp higher-order repeat unit is completely identical within all the arrays of the different chromosome ends, which further explains the uniform increments in the TRF ladder pattern.

**Fig. 6:**
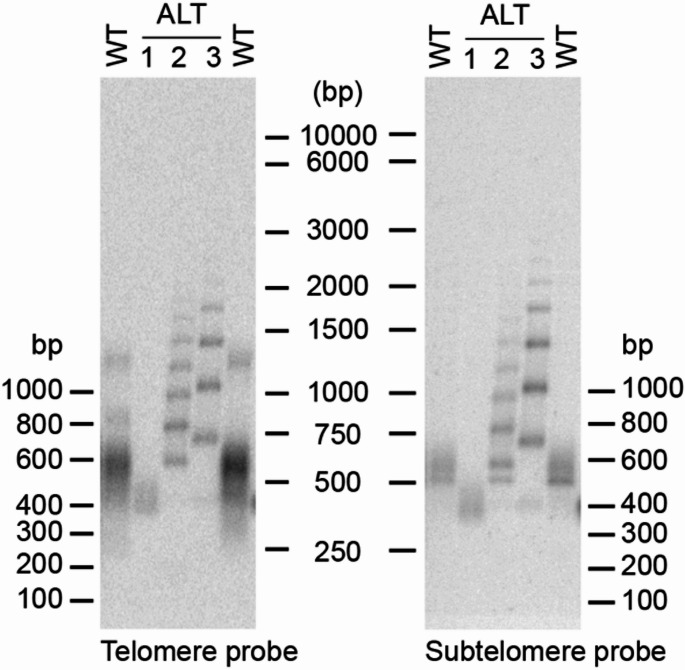
Differences in the ladder pattern increments between strains indicate that differently sized repeat units are used for the elongations. TRF assay of three separate ALT strains show differences in band patterns. WT: YMC48 (left) and Y235 (right). ALT strain #1; YMC482 (S3), #2–3; YMC133 (S4 and S10). The membrane was hybridized successively with a telomere (left) versus a subtelomere probe (right)

However, intriguingly, we have observed samples exhibiting ladder patterns with different size increments, implying that the arrays in those strains consist of differently sized repeated elements (Fig. 6). Significantly, the band increments show a consistency within each sample, underlining the notion that the same specific element is being added onto the telomeres within the respective separate populations of established ALT cells.

In conclusion, the summarized results from the genome sequencing, TRF assays and Telomere-PCR analyses show a convergent sequence in all the subtelomeres of the ALT cell chromosomes, where one specific TelKO element variant is predominantly spread onto all telomeres in each separate ALT strain establishment. Hence, the TelKO element is a key for the homogenization and stabilization of the shortened telomeres.

### The telomere protein Rap1 can bind to TelKO elements, suggesting formation of protective chromatin caps on the short ALT telomeres

**Fig. 7:**
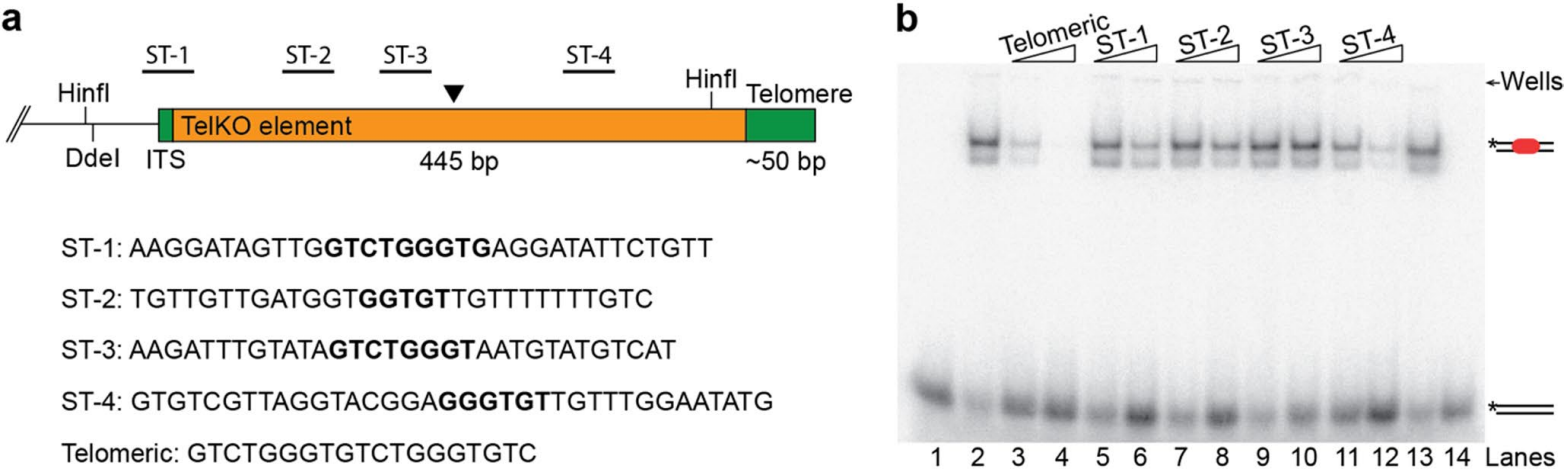
The telomere protein Rap1 binds with low affinity to the TelKO element. Competition Electrophoretic Mobility Shift Assay (EMSA) of Rap1 incubated with a labeled telomeric probe and increasing amounts of the non-labeled oligonucleotide competitors ST-1-4. (**a**) Schematic of the subtelomeric region with indicated positions and sequences of the subtelomeric regions tested. Interstitial telomeric sequences are indicated in bold. (**b**) EMSA gel where the Rap1 binding to the labeled telomeric probe was competed by the addition of non-labeled subtelomeric competitors added in 400 and 4000 times molar excess (lanes 5–12). As a control, the non-labeled telomeric probe was added in 40 and 400 times excess (lanes 3–4). Lanes 1 and 14, no protein added. Lanes 2 and 13, no competitor added. The positions of the free DNA versus the DNA-protein complex are indicated to the right

Canonical telomeric sequences show a characteristic strand bias, with a TG-rich strand running 5’-3’ towards the end. Interestingly, the sequence of the TelKO element also shows a slight TG-rich strand bias. The TelKO_445_ has an overall [T + G] content of 75%, with its first part, corresponding to the TelKO_220_ variant, having an elevated [T + G] content of 82%. This is emphasized by the presence of several short Interstitial Telomeric Sequences (ITS) that are scattered along the element, mostly only partial stretches of the *N. castellii* telomeric sequence (TCTGGGTG). Since the telomere binding protein Rap1 has previously been demonstrated to show a spatial flexibility and is able to bind to different variant sequences, we reasoned that it might accommodate binding to the TG-rich sequences in the TelKO element (Wahlin and Cohn 2000, 2002). To learn more about the functionality of the TelKO element, we therefore decided to analyze whether Rap1 would be able to bind to the subtelomeric TG-rich stretches in vitro. We designed ds-oligonucleotides distributed along the TelKO region (ST-1 – ST-4) (Fig. 7a). ST-1 is centered around a 9 bp ITS that marks the border of the TelKO element and ST-3 covers an 8 bp ITS close to the end of the TelKO_220_ variant. Although ST-2 only contains a 5 bp ITS, it is overall highly TG-rich, and ST-4, specific for the TelKO_445_ variant, incorporates a 6 bp ITS surrounded by a highly TG-rich sequence.

In an Electrophoretic Mobility Shift Assay (EMSA), Rap1 produced the expected shifted DNA-Rap1 complexes, showing that Rap1 can bind to the subtelomeric oligonucleotides, although with a quite low affinity compared to the control oligonucleotide containing a fully telomeric sequence (data not shown). To quantitatively assess the binding capacity to the different sequences, we performed EMSA competition assays (Fig. 7b). Here, the binding of Rap1 to the radiolabeled telomeric oligonucleotide was challenged by adding non-labelled ST oligonucleotide competitors in large molar excess. Addition of the non-labelled telomeric sequence effectively competes away the shifted band, leading to an increase of the free DNA already at 40x molar excess (Fig. 7b, lanes 3–4). The non-labelled ST oligonucleotides also exhibit competition, but a higher molar excess is needed (400x-4000x), indicating a much lower affinity of Rap1 to all these subtelomeric sequences (lanes 5–12). Quantitation of the shifted bands at 400x subtelomeric competitor, shows around 30–90 times higher signal left in the shifted band when compared to the telomeric sequence (Table S1). Notably, ST-4 has the highest affinity among the subtelomeric sequences tested, despite containing only a 6 bp ITS. This result opens for the possibility that other TG-rich stretches in the TelKO element could be possible targets for Rap1 binding.

Intriguingly, the presence of Rap1 binding sites within the TelKO element suggests that the subtelomeric region might be incorporated within the telomeric chromatin in the ALT cells. Although we cannot exclude the possibility that the shortened telomeres may be stable independently in *N. castellii*, accumulated data in various species show that short telomeres are highly unstable. We therefore hypothesize that the low affinity binding sites may become exposed and accessible for Rap1 when the telomeres shorten, thereby providing a protective cap structure and stabilizing the chromosome ends.

## Discussion

### ALT cells maintain short and stable telomeres

The budding yeast *N. castellii* shows a remarkably effective switch to the ALT mechanism and is able to maintain chromosome integrity and bypass senescence after telomerase disruption (Cohn et al. 2019). Here we showed that the subtelomeric region just internal to the telomere, named the TelKO element, plays a key role in the stabilization of the telomeres in the ALT cells. In the wild-type strain (WT), the TelKO element resides exclusively in the subtelomeres of a subset of the telomeres, located immediately adjacent to the ∼320 bp telomeric sequence. We found that the TelKO elements are present in two main variants in the WT strain, where the 220 bp element (TelKO_220_) is a shorter version of the long 445 bp element (TelKO_445_). In addition, some shorter TelKO_445_ variants were detected, which extend a bit further beyond the TelKO_220_ border. Notably, like previous findings regarding *S. cerevisiae* Y’ elements, only the internal part of the TelKO element was observed as degenerate shorter variants, not the distal part (Louis et al. 1994).

Our TRF assays of the serial plate streaking show that the telomeres of the telomerase-deficient strains rapidly shorten and reach a short terminal structure that is stable throughout the subsequent streaks. The presence of a single focused band in the TRF assay is a striking characteristic, which is indicative of a uniform structure at the chromosome termini. Significantly, it indicates that telomeres are kept at a more tightly regulated length compared to the variable lengths in the WT telomeres. Here we analyzed the telomere structure in populations of clones within the respective streaks, which showed that all the clones keep the same short structure. This implies that the initial structure that is established in a particular cell is stabilizing the telomeres genome-wide, and that this stable structure is then passed on throughout excessive numbers of generations. It is noteworthy, that the lack of dynamic rearrangements points to a functional capping of the telomeres.

Most of the ALT strains exhibit a short band size at ∼350 bp, corresponding to a terminal structure consisting of the TelKO_220_ element followed by ∼50–60 bp telomeric repeats. This short telomere structure seems to be prevailing in the ALT cells, with only a low frequency of strong ladder patterns appearing, implying massive elongation events. Notably, in some serial streaking experiments no traces of any ladder patterns can be observed in any of 16 subsequent streaks.

Subtelomeric regions generally contain vast numbers of repetitive sequences and are dynamic in nature (Cohn et al. 2006; Snoek et al. 2014; Garcia-Rios et al. 2017). In the sequenced *N. castellii* WT strain, a single TelKO element is present in the respective chromosome end. However, the TelKO element contains several interstitial telomeric sequences (ITS) as well as short TG-rich tandem repeats, a characteristic feature of subtelomeric regions immediately adjacent to the telomeres (Suppl. Fig. S1). As repeated sequences are prone to take part in recombinational activities, it is surprising that this region is kept as a highly conserved entity among the strains, devoid of any reorganization or amplification of the repeated sequences. It is therefore tempting to speculate that the specific structure of the TelKO element confers a stability to the WT subtelomeres. This leads us to hypothesize that the subtelomere stability might become an asset when the telomeres are shortening in the ALT cells. It is noteworthy that the *N. castellii* ALT cells show no trace of type II-like events, a feature that could be dependent on the nature of the DNA sequences present in the subtelomeres. In *S. cerevisiae*, it has been demonstrated that different subtelomeric elements can influence the selection of the recombinational pathway used for telomere maintenance (Grandin et al. 2020). On the other hand, the choice of ALT pathway could also be dependent on the genetic constitution of this species.

### Telomeres are elongated by homogeneous tandem arrays of subtelomeric elements

Telomeres have been shown to form a loop structure (t-loop) in human cells and many other species, which is suggested to contribute to stability and protection of the DNA ends, as well as to self-primed telomere extension (Tomaska et al. 2019). T-loops are correlated to long telomeres, and they were observed in *Kluyveromyces lactis* cells manipulated to have abnormally elongated telomeres (Cesare et al. 2008; Jones et al. 2023). Intrachromosomal recombinational activities at t-loops may lead to release of extrachromosomal circular molecules consisting of telomeric DNA (ECCs), which can consequently cause rapid shortening of the telomeres (Tomaska et al. 2019; Jones et al. 2023; Xu and McEachern 2012; Rivera et al. 2017). Specifically, ECCs consisting of the telomeric C-strand (C-circles) are regarded as a hallmark of human ALT cells and have also been detected in *S. cerevisiae* (Cesare and Reddel 2010; Aguilera et al. 2022). A spectrum of different inter- and intra-chromosomal BIR mechanisms have been suggested for the recombination-mediated lengthening of telomeres in ALT cells, and ECCs are implicated as a source of template used in the rolling-circle replication mechanism (Tomaska et al. 2019; Cesare and Reddel 2010; Natarajan and McEachern 2002).

Although the *N. castellii* ALT telomeres predominantly exhibit a short structure, a few samples show a ladder pattern in the serial passaging, implying stochastically occurring telomere elongation events. Hypothetically, this may indicate occasional loss of telomere capping, maybe due to gradual shortening of the telomeres, leaving them uncapped and unstable. Many different recombinational mechanisms may be acting to create long arrays of repeats using homologous recombination (HR)-mediated telomere extension by break-induced replication (BIR) (Pickett and Reddel 2015; Xu and McEachern 2012). As previously suggested, the strand invasion could be the result of either a telomeric intra- or interchromosomal annealing of the short telomere, using a longer telomere as a copy template (Pickett and Reddel 2015; Xu and McEachern 2012; Cesare and Reddel 2010). Alternatively, extrachromosomal circular DNA templates (ECCs) could be used in a rolling-circle amplification (Pickett and Reddel 2015; Xu and McEachern 2012; Cesare and Reddel 2010). In *N. castellii*, the TelKO elements contain short ITSs that could be targets for the strand invasion of a short telomere. Specifically, when targeting the flanking ITS upstream of the TelKO element (ST-1), this would enable the copying of the full TelKO element. We presume that an intrachromosomal strand invasion would more easily produce long repeated arrays of the same identical sequence block. However, we cannot rule out the possibility that the ladder pattern is produced by the appearance of ECCs, which can be used in a rolling-circle mechanism to rapidly elongate telomeres with long arrays of identical repeats (Natarajan and McEachern 2002). In the so-called roll-and-spread model, it was suggested that a long array generated from a telomere extension event can then spread to other telomeres via BIR, resulting in identical arrays on different telomeres (Xu and McEachern 2012; Natarajan and McEachern 2002). Our genome sequencing data is consistent with such a mechanism, as the identical composite repeat is residing in all chromosome ends (Fig. 5).

Interestingly, in a few of the serial streaking assays, we observed a shift in the TRF band size, which was explained by a change in the subtelomere structure to encompass the TelKO_445_ element (Fig. 1c). Since the band shift is very clearcut, this would indicate a quite rapid genome-wide replacement of the predominant subtelomeric structure. As previously suggested by Xu & McEachern (2012), this could be due to the occurrence of a concerted action of array amplification and replacements, where a secondary rolling-circle amplification event generates a new array, which is followed by stochastic BIR events copying the new array onto other telomeres (Xu and McEachern 2012). We believe that we might have caught this mechanism in action, as a ladder pattern appears in the streak in between the two separate streaks having the TelKO_220_ (S3) versus the TelKO_445_ (S5) elements (Fig. 1c). The ladder pattern (S4) may therefore indicate a dynamic phase which finally ends up in the replacement of TelKO_220_, through spreading of TelKO_445_. To verify this model, it would be very interesting to investigate whether we have the occurrence of ECCs containing the TelKO element. In theory, the new repeat array could be created by intra-chromosomal elongation of a remnant TelKO_445_ telomere. Such a mechanism could involve a telomere loop formation and 3’ end invasion of the upstream subtelomeric region to use it for repeated self-copying and extension. Regardless of the underlying mechanism driving the observed activity, the rapid and prominent concerted replacement process is quite intriguing, especially given the absence of any imposed selective advantage on the cells.

### Telomere trimming cause drastic reductions of subtelomeric arrays

In the serial streaking assays of *N. castellii*, the appearance of a ladder pattern in one streak is commonly observed to shift back to the short band in the following streak (Fig. 1b). This feature would presumably involve some kind of telomere trimming event. Indeed, hyper-extended telomeres have been reported to undergo trimming that rapidly shorten the length and induce accumulation of telomeric circles (Jones et al. 2023; Xu and McEachern 2012; Rivera et al. 2017). The mechanism termed telomeric rapid deletion (TRD) is proposed to involve the cleavage of a telomeric loop formed by the strand invasion of a single-stranded telomeric 3’ end into the internal double-stranded part of the same telomere (Xu and McEachern 2012). Moreover, internal loop structures (i-loops) have been identified in human ALT telomeres, which are induced by single-stranded damage in the telomeric sequence, and which cause telomere shortening when excised as telomeric circles (Mazzucco et al. 2020). We reason that, when the subtelomeric TelKO elements become terminally positioned in the *N. castellii* chromosome ends, similar rapid and large deletion events may take place for the TelKO arrays.

On the other hand, although sporadic lengthening events occur in *N. castellii*, most clones exhibit a short and distinct structure which is kept stable through extensive passaging. It is plausible that this involves very active trimming mechanisms that immediately shorten any appearing elongated array. Hypothetically, if the extension mechanism occurs by intra-chromosomal strand invasion forming a loop structure (t-loop), the t-loop might also be a substrate for the trimming event. Formation of an intermediate t-loop structure that could act as the basis for alternate extension and trimming activities would be a highly effective way to obtain the observed telomere length homeostasis in *N. castellii*.

### Arrays of uniform repeated elements imply continuous telomere elongation events

The homogenization of the telomeres is evident in the Telomere-PCR results, as we obtain distinct bands and a good resolution in the DNA sequencing in all ALT strains. Notably, in this method we only retrieve information about the innermost subtelomeric element, since the yield from longer repeat array products is too low to obtain productive sequencing results. However, the ladder patterns observed in the TRF assays provide useful additional information about the array structure. The fact that the bands always exhibit uniform increments indicates that the same element is repeated within the array. The genome sequence data confirms this notion for one of the strains, showing that the exact same 325 bp composite repeat block is added in the array. This higher-order repeat consists of the TelKO_220_ element and specific flanking sequences including identical telomeric stretches (Fig. 5). Remarkably, this indicates that the elongation mechanism is repeatedly using exactly the same sequence as a template for the extension.

The higher-order repeat in the sequenced ALT strain is rather complex and we have not been able to find any simple route to explain its formation. It is worth noting that the TelKO sequence located just before the 13 bp segment integrated in the higher-order repeat, has a 2 bp telomere sequence homology (TG), which could indicate a mechanism including microhomology-mediated BIR. However, since we discovered ladder patterns showing a difference in the incremental band size, we believe that other types of complex composite blocks can also form, composed of different sub-parts (Fig. 6). As seen in Fig. 6, the ladder bands of both samples contain the TelKO element, while the difference in band size suggest that different kinds of flanking sequences are included in the repeated block. Notably, the uniform ladders in the respective sample indicate that the repeats are homogeneous. Hence, we conclude that, independent on which higher-order block that is used for ALT telomere extension, the array seems to be produced by a continuous mechanism, giving rise to homogeneously repeated arrays. To elucidate the mechanism for the establishment of the composite blocks, it will be highly interesting to expand the sequencing approach to include more strains. Since the spreading of uniform repeats resembles the ECC-based roll-and-spread mechanism described in *K. lactis*, we would like to investigate whether ECCs can be discovered in the *N. castellii* ALT cells and whether they have structures explaining these intriguing phenomena (Natarajan and McEachern 2002).

### Incorporation of a subtelomeric region in the protective chromatin cap can provide stability to short ALT telomeres

The conformity of the telomere structure in *N. castellii* ALT cells and the stable transfer through extensive numbers of generations are extraordinary features. This stability could be based on the ability of Rap1 to bind to the TG-rich sequences in the TelKO element. We found that Rap1 can bind with low affinity to four regions distributed along the TelKO element (ST-1-4). Although these regions contain telomeric sequences, the respective ITS is too short to form a proper binding site, since *N. castellii* Rap1 has a 12 bp telomeric minimal binding site (Rhodin Edsö et al. 2011). Surprisingly, Rap1 showed the highest binding to ST-4, which contains only a 6 bp ITS. However, Rap1 has been shown to be able to adapt to variant target sequences by its spatial flexibility property (Wahlin and Cohn 2000, 2002). Therefore, we speculate that the binding is accommodated by the help of TG-rich sequences flanking the ITS, implying that Rap1 interaction probably could be established also in other parts along the TG-rich TelKO element.

We contemplate whether the low affinity binding sites in the TelKO elements are able to attract Rap1 molecules in the cellular context. However, when the telomeric sequences are shortening and hence the high affinity binding sites are reduced, we envision that Rap1 would be able to get access to the TelKO element. In fact, since Rap1 has been shown to bind cooperatively, the binding to the TelKO element could be stabilized by the Rap1 molecules binding to the remaining short telomeric stretch. In this way, incorporation of the TelKO element into the terminal chromatin structure would enable enough Rap1 proteins to recruit other protective protein partners, leading to formation of a protective telomere cap. In this line, we note that our previous analyses showed the presence of telomeric single-stranded 3’ overhangs in *N. castellii* ALT strains, having a similar preferred 5’ permutation as in wild-type cells, thus further corroborating the possibility of cap formation (Itriago et al. 2022; Fridholm et al. 2013).

Putatively, the weak Rap1 binding affinity to TelKO sequences could be a key to the ALT mechanism, since a too high binding affinity would produce a compact chromatin structure that would presumably repress recombination. Inevitably, continued cell division will lead to attrition of the remnant telomeric sequences, which would trigger recombinational lengthening events in a continuous turnover of the terminal TelKO element. We hypothesize that the terminal stretch of telomeric sequences may be replenished as part of this recombination process, possibly in combination with a terminal trimming event.

Together, our data show that the maintenance of the *N. castellii* ALT telomeres is provided by specific subtelomeric sequences that are added to the DNA ends, leading to homogenization and stability of short telomeres, and avoiding gross genomic rearrangements. The rapid spreading of TelKO elements to all telomeres effectively rescues the cell division capacity, enabling the cells to bypass any major growth crisis after loss of telomerase. It will therefore be interesting to further investigate the underlying molecular mechanism for the distribution of the elements. Since the TelKO element variants can be used as markers to analyze the dynamics of the spreading, as naturally occurring telomere tags, we anticipate that studies of the budding yeast *N. castellii* will bring important insights into the shaping of ALT telomeres.

## Supplementary Information

Below is the link to the electronic supplementary material.

Supplementary Material 1

## Data Availability

No datasets were generated or analysed during the current study.
