# Supplementary material for: Subtelomeric elements provide stability to short telomeres in telomerase-negative cells of the budding yeast Naumovozyma castellii
Source: Curr Genet. 2025 Sep 3;71(1):19. doi: 10.1007/s00294-025-01325-w (PMC12408736; doi:10.1007/s00294-025-01325-w)
Supplement: Supplementary file 1 — Supplementary Material 1 [file 294_2025_1325_MOESM1_ESM.pdf]

Supplementary information
Current Genetics

Article title:

Subtelomeric elements provide stability to short telomeres in telomerase-negative cells of the budding yeast *Naumovozyma castellii*.

Authors:

Rishi K. Jaiswal (1), Teresa Garibo Domingo (1), Héloïse Grunchec (1), Komudi Singh (2), Mehdi Pirooznia (2), Eran Elhaik (1), Marita Cohn (1)*

*corresponding author: marita.cohn@biol.lu.se

Author affiliations:

(1) Department of Biology, Lund University, Sölvegatan 35, SE-223 62 Lund, Sweden.

(2) Bioinformatics and Computational Core Facility, NIH, Bethesda, Maryland, USA.

Supplementary figures

a

AGGATATTCTGTTTATGTTTATGTTTATGTTTATCTTTGTTTGTTTGTTTATTTGTGT
GTTTGTTTTTGTTTGTGTATGTTGGAGGTATTTGTTGTTGATGGTGGTGTTGTTTT
TTTGTCTTTGTGTTTTTTTTCTGGGAGGTGGTTGGTGGATCTAAGATTTGTATAGT
CTGGGTAATGTATGTCATGGGGTACGAGAAAATGTTGTCTTGGTGAAATTTTAGG
GGAGCGCGATGCGTGCTTATATAATCGGTTGGTCTGAGGTTGGTTGGTGGGAGA
AAGTGTCGTTAGGTACGGAGGGTGTTGTTTGGAATATGGTGGGATGGAATAGGT
AGTATTTTCAAGGAATTGGTGGCCGCGTGGTGCAGCGGTTGTCGGGGTTGCTT
GTATGAGCGGTCTCGGAAAGAGCCGGGTTCGAATCCCGGGGCGATCAAGAGGT
TCCTTA

HinfI

b

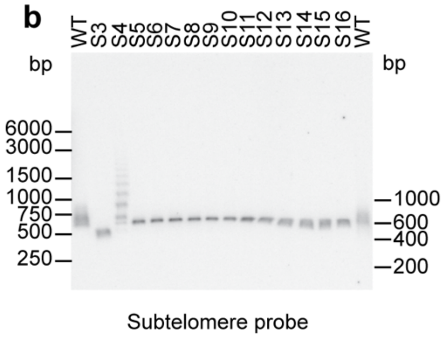

Fig. S1 Discovery of two TelKO element variants. (**a**) Sequence of the subtelomeric $TelKO_{445}$ element in *N. castellii*. The $TelKO_{220}$ element is a truncated version of the $TelKO_{445}$ element, indicated by the red arrowhead. The terminal HinfI site in the $TelKO_{445}$ element is indicated by a grey box. The WT strain contains two alleles of the $TelKO_{220}$ element, differing by having either T or C at position 28 (blue box). (**b**) Rehybridization of the TRF assay membrane in Fig. 1D with the universal subtelomeric probe shared by both TelKO element variants. Serial streaking assay of YMC133 (*tlc1*$^-$/ *tlc1*$^-$, S3-S16)

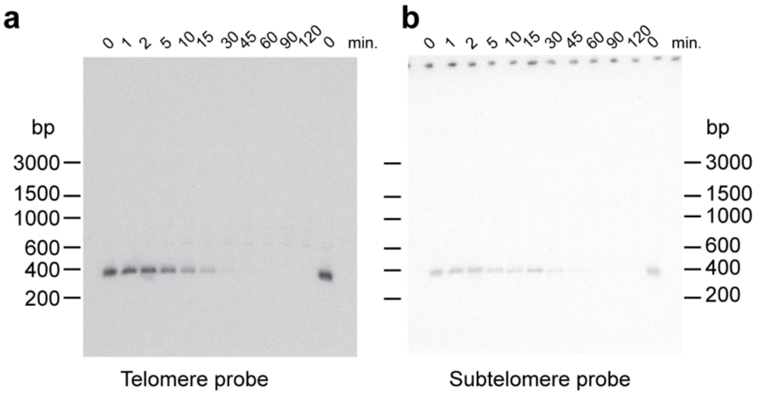

Fig. S2 BAL-31 assays show that the TelKO elements remain terminally located in the ALT strains. Genomic DNA of the ALT strain (YMC133) was cleaved with BAL-31 for increasing periods of time (0-120 min, as indicated above the gel), followed by the TRF assay procedure using HinfI digestion. The membrane was hybridized successively with a telomere probe (**a**), versus the universal subtelomere probe shared by both TelKO element variants (**b**)

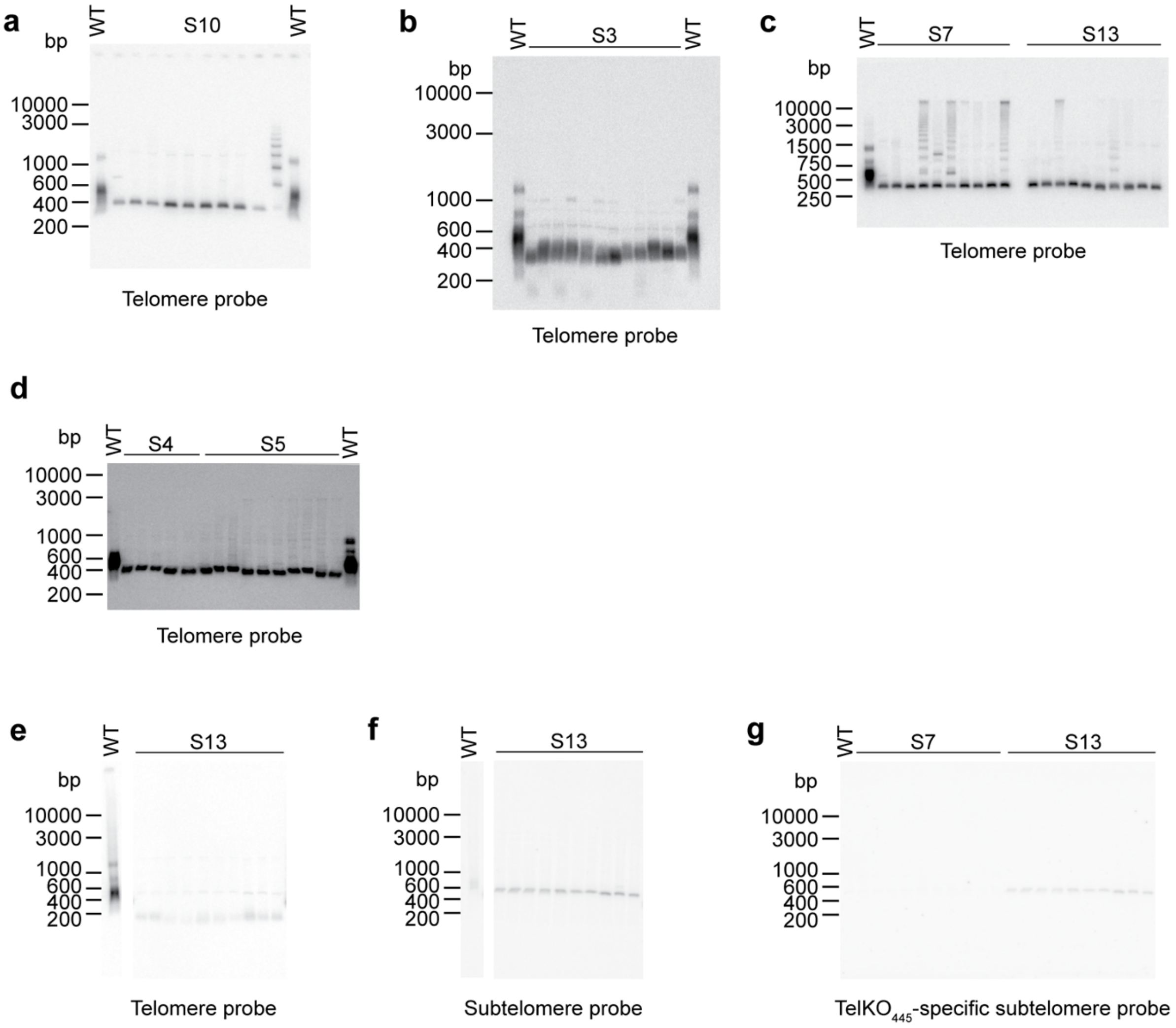

Fig. S3 Large populations of ALT colonies show homogeneous structures both within and between different streaks. TRF assays using HinfI digestion were performed on ten separate colonies isolated from each of the indicated streaks of different ALT strains. (**a**) YMC133 (*tlc1*$^-$/ *tlc1*$^-$, S10); WT, Y235. (**b-c**) YMC482 (*tlc1*$^-$, S3, S7, S13); WT, YMC48. (**d**) A long exposure of YMC133 (S4, S5) shows traces of ladder patterns, indicating low frequencies of elongation events. (**e-g**) The membrane of YMC133 (S13) was first hybridized with the telomere probe (**e**), and subsequently hybridized with the subtelomere probe shared by both TelKO element variants (**f**), followed by a probe specific for the TelKO$_{445}$ variant (**g**). (**g**) Two different streaking events of the YMC133 (S7 versus S13 samples) show the establishment of either the short TelKO$_{220}$ variant (S7 samples) or the long TelKO$_{445}$ variant (S13 samples), in the respective strains. The S7 samples lack signals, since only the TelKO$_{445}$ variant is detected by the TelKO$_{445}$-specific probe used here

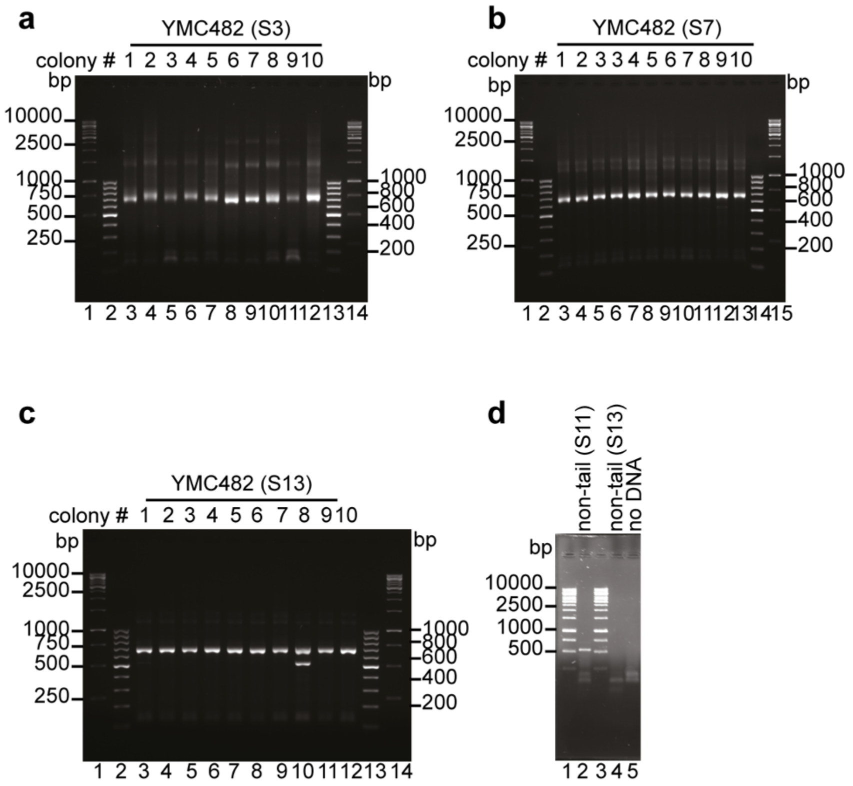

Fig. S4 Telomere-PCR generates homogeneous amplicons of the terminal ends in populations of ALT colonies. Telomere-PCR was performed on C-tailed genomic DNA from ALT cells of YMC482 from subsequent streaks (S3, S7, S13). (**a-c**) An amplicon band of ~700 bp is generated in all streaks, indicating the presence of the $TelKO_{220}$ variant. (**d**) Telomere-PCR control reactions of YMC133 (S11, S13); Lane 5, no DNA added; lanes 2 and 4, no C-tailing performed. Non-specific background bands stochastically occur in the reactions (**d**, lane 2 and **c**, lane 10)

a

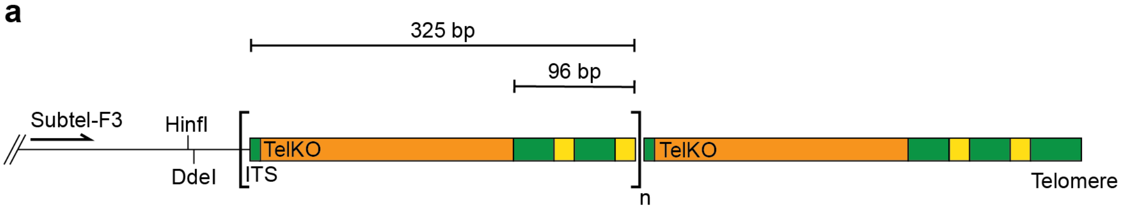

b

GTCTGGGTGAGGATATTCTGTTTATGTTTATGTTTACGTTTATCTTTGTTTGTTTGTTTATT
TGTGTGTTTGTTTTTGTTTGTGTATGTTGGAGGTATTTGTTGTTGATGGTGGTGTTGTTT
TTTTGTCTTTGTGTTTTTTTTCTGGGAGGTGGTTGGTGGATCTAAGATTTGTATAGTCTG
GGTAATGTATGTCATGGGGTACGAGAAAATGTTGTCTTGGTGAAATTGTCTGGGTGTCT
GGGTGTGTCTGGGTGTCTGGGTGTCTTGGTGAAATTGTCTGGGTGTCTGGGTGTGTC
TGGGTGTCTGGGTGTCTTGGTGAAATT

Fig. S5 All telomeres of an ALT strain contain the same composite higher order repeat. (**a**) Schematic overview (same as Fig. 5) of the sequence found at all telomeres of the ALT strain YMC481 (*tlc1*$^{-}$, S5). The number of 325 bp higher order repeats vary between 2-27 (denoted by n), and they are mainly identical, containing only a small number of single nucleotide variations. (**b**) DNA sequence of the higher order repeat. The 325 bp higher order repeat contains the $TelKO_{220}$ variant, flanked internally by a short 9 bp interstitial telomeric sequence (ITS, green box) and distally by a 96 bp element containing both telomeric and TelKO sequences. The 96 bp element contains two segments of identical 35 bp telomeric sequences (green), each followed by a 13 bp sequence corresponding to the end of the $TelKO_{220}$ element (yellow). Hence, the terminal 13 bp of $TelKO_{220}$ is represented in three copies in this composite structure

Competitor	Ratio ST:telomeric sequence
Telomeric sequence	1 ± 0
ST-1	60 ± 14
ST-2	66 ± 13
ST-3	91 ± 13
ST-4	33 ± 7

Table S1 The telomere protein Rap1 binds with lower affinity to the subtelomeric TelKO element compared to the telomeric sequences. Quantification of the competition EMSA (Fig. 7). The percentage of bound probe (% shifted band) was determined by dividing the signal of the shifted band by the total signal (shifted plus unshifted) in the respective lanes containing 400x molar excess competitor. The ratio of the subtelomeric (ST-1-4) and telomeric values were calculated (ST:telomeric sequence) to obtain the fold difference. Mean values and standard deviation (SD) are presented for the technical replicates (n=5). As a lower ratio indicates a higher binding affinity, the results show that Rap1 has the highest binding affinity to ST-4
